# Primary Care Providers Describe Barriers and Facilitators to Amputation Prevention in Oklahoma

**DOI:** 10.3390/jcm14196817

**Published:** 2025-09-26

**Authors:** Austin Milton, Dana Thomas, Freddie Wilson, Blake Lesselroth, Juell Homco, Wato Nsa, Peter Nelson, Kelly Kempe

**Affiliations:** 1 School of Community Medicine, University of Oklahoma, 4502 E. 41st St., Tulsa, OK 74135, USA; austin.milton@hsc.utah.edu (A.M.); dana-thomas-1@ou.edu (D.T.);; 2 Department of Medical Informatics, School of Community Medicine, University of Oklahoma, 4502 E. 41st St., Tulsa, OK 74135, USA; blake-lesselroth@ou.edu (B.L.); juell-homco@ou.edu (J.H.); wato-nsa@ouhsc.edu (W.N.); 3 Division of Vascular Surgery, Department of General Surgery, School of Community Medicine, University of Oklahoma, 4502 E. 41st St., Tulsa, OK 74135, USA; peter-nelson@ou.edu

**Keywords:** amputation, diabetes, limb preservation, limb salvage, peripheral artery disease, prevention

## Abstract

**Background**: Although most amputations caused by diabetes and peripheral artery disease (PAD) are preventable, current limb preservation efforts in the United States remain poorly understood. This study aims to identify key barriers and facilitators to limb preservation from the primary care provider (PCP) perspective. We plan to use the insights from this work to promote targeted intervention strategies. **Methods**: Using a mixed-methods design, an online 5–10 min survey was distributed to Oklahoma primary care providers who could elect to participate further in a semi-structured, audio-recorded interview. Descriptive analysis was used to summarize survey results. Interviews were transcribed and qualitatively analyzed using grounded theory. Donabedian’s structure, process, and outcome framework was used to categorize how each identified barrier and facilitator increases or reduces the risk of limb loss for at-risk patients at the practice level. Finally, we compared and contrasted survey and interview findings. **Results**: Thirty surveys were completed (approximately 14% response rate), and seven interviews were conducted with PCPs geographically dispersed across Oklahoma. Most clinicians reported in the survey that they see at-risk limbs at least once every 1–2 months (*n* = 29, 96.7%). Half of clinicians were satisfied or very satisfied with access to vascular surgery (*n* = 15, 50.0%), interventional specialists (*n* = 13, 43.3%), and endocrinologists (*n* = 12, 40.0%). Finally, survey respondents reported that social needs most often affecting their patients with a limb at risk of amputation include income, health education, transportation, and health insurance. Interviews confirmed PCPs frequently see at-risk limbs. We identified thematic barriers to limb preservation that included limited access to specialty care, limited PCP and patient amputation prevention education, and patient social struggles surrounding transportation, finances, and insurance. Patient advocates (community, clinical, or personal), affordable medications, and more time with patients were reported as facilitators in amputation prevention. **Conclusions**: Oklahoma PCPs frequently see at-risk feet, realize poor access to care, and desire structural change to support excellent preventive care in diabetes and PAD. Limb preservation in Oklahoma is contingent upon shifting from disempowerment to engagement that requires systemic reform, clinical innovation, and community engagement. We identified several intervention strategies, including increasing education for PCPs to empower them to initiate early prevention, improving early identification and preventive therapy for patients at risk for limb loss, and cultivating specialty care access via networking and policy change.

## 1. Introduction

Chronic disease-related lower extremity amputations (LEAs), primarily resulting from advanced diabetes and/or peripheral artery disease (PAD), are an urgent health concern, considered preventable, and deserve critical study. LEAs are rising in high-risk subgroups within the United States, the highest of which include those from specific geographic regions, of a lower socioeconomic status, or Black or American Indian race [1]. Following a major amputation (above- or below-the-knee), there is a much higher risk of mortality, morbidity, and immobility that results in substantial personal and societal costs [2]. Annual US healthcare dollars spent during and after a major amputation are estimated to be between 80 and 300 billion dollars [1].

### 1.1. Peripheral Artery Disease and Chronic Disease-Related Amputations

PAD is defined as a narrowing or blockage of the peripheral arterial tree and occurs most frequently in the lower extremities. It is extremely common, with an estimated 8–12.5 million in the United States [1]. PAD in individuals is often unrecognized as atypical leg pain, and one may go for years without a known diagnosis, until their limb may be overtly threatened [3]. Chronic limb-threatening ischemia is the end-stage of PAD, defined by clinical presentation of rest pain, tissue loss, or gangrene due to severely narrowed or obstructed arterial disease. It requires urgent or emergent revascularization, wound care, and/or infection control. Unfortunately, limb amputation can result when these interventions are delayed or if the disease has progressed beyond repair [4].

### 1.2. Peripheral Artery Disease and Diabetes

Over the past 30 years, the global prevalence of diabetes has approximately doubled, and PAD has risen by 72 percent [5]. Similarly, in the United States, diabetes and PAD prevalence are rapidly rising [1]. While there is a strong association with diabetes as to the cause of rising rates of PAD in the United States, the prevalence of PAD risk factors, independent of diabetes, also continues to climb (including hypertension, hyperlipidemia, chronic kidney disease, central obesity, physical inactivity, and advanced age) [6]. Importantly, patients with concomitant diabetes and PAD have a multiplicative risk for complications, with some estimating a fourfold risk increase in amputation [1,2,3,7,8,9,10]. It is unlikely that the need for diabetes or PAD LEA-prevention initiatives will decrease soon.

### 1.3. Chronic Disease-Related Amputations Are Preventable

Nevertheless, LEAs are considered preventable. At a much earlier stage in a person’s disease risk and disease lifecycle, prevention from limb loss can occur through a healthy whole food diet, walking therapy, behavioral modification, and best medical therapy [11]. Much is written about diabetes-related amputation prevention, but there are far fewer initiatives on PAD-related amputations [12]. Even less is known about the perspectives of frontline professionals, particularly primary care providers (PCPs), who play a crucial role in chronic disease-related amputation prevention [6]. But research and efforts are beginning to acknowledge and combat these increasing rates, and a contemporary multispecialty amputation prevention policy statement recommends a multipronged initiative [2,12,13,14].

### 1.4. Local Multidisciplinary Work

Before our multidisciplinary team began our work in amputation prevention in Oklahoma seven years ago, there was no ongoing active federally funded limb preservation research in our state or region. As a starting point, we analyzed national amputation overviews and Oklahoma discharge data, identifying contemporary challenges. We found Oklahoma to have one of the leading upward trends in amputation rates in the United States [2,15]. At a state level, over 1000 limbs were amputated per year, between 2008 and 2019, and amputation rates have nearly doubled over the past decade [2]. Today, despite rising amputation rates amongst subgroups (such as our geographical state), there is limited national understanding of why some subgroups are at higher risk and what effective preventive measures we should be using to combat them.

### 1.5. Study Objective

Chronic disease-related amputations are an increasingly prevalent yet preventable public health issue that remains insufficiently explored in current research and prevention initiatives. This study’s objective was to identify from primary care providers’ perspectives the barriers and facilitators contributing to limb preservation in Oklahoma. After creating a new conceptual model derived from this study, we plan to target the management of diabetes and peripheral artery disease to find new approaches for limb preservation.

## 2. Materials and Methods

### 2.1. Overview

To gain a comprehensive understanding of Oklahoma amputation prevention from the PCP perspective, we utilized a convergent parallel mixed-methods design [16]. An online 5–10 min survey was distributed to PCPs across Oklahoma and quantitatively analyzed. At the end of the survey, PCPs could elect to participate further in a semi-structured, audio-recorded 45–60 min interview. Interviews were transcribed and qualitatively analyzed using grounded theory. For further healthcare quality evaluation, we used Donabedian’s structure, process, and outcome framework to categorize how each identified barrier and facilitator contributes to increasing or reducing the risk of limb loss for at-risk patients. We merged the results from both analyses to compare, contrast, and combine the findings. The creation and approval of survey and interview questions were overseen by our multidisciplinary team, comprising research and clinical experts in epidemiology, biostatistics, medical informatics, dissemination and implementation sciences, vascular surgery, internal medicine, and podiatry. Creation of the survey and interview questions was performed by four authors. This included two vascular surgeons (KK and PRN), one physician with internal medicine and implementation expertise (BL), and one epidemiologist and implementation expert (JH). Further editing and approval was performed by our podiatry and biostatistical experts. In addition, Oklahoma Primary Healthcare Improvement Cooperative (OPHIC) leadership edited and approved the survey and interview questions. The semi-structure interview was piloted with our researcher in internal medicine as the interviewee. The study was conducted in accordance with the Declaration of Helsinki, and the protocol was approved by the University of Oklahoma Health Sciences Center Institutional Review Board (12773) on 22 January 2021. 

### 2.2. Surveys

A REDCap™ survey request was emailed to over 200 Oklahoma primary care clinicians in partnership with OPHIC. Informed consent for participation was obtained from all subjects involved in the study. OPHIC disseminates and implements evidence-based practices for primary care and has shared a database of practices with high rates of cardiovascular disease for survey distribution. Survey participants signed a consent and attested to practicing as a licensed Physician Assistant, Nurse Practitioner, Medical Doctor, or Doctor of Osteopathic Medicine prior to beginning the questionnaire. Question structure included binary, Likert scale, ranking, and open-ended formats. The survey had 18 questions, covering eight topics (Table 1). The complete survey is supplied (Supplementary S1). We calculated descriptive statistics for all survey responses using Microsoft Excel for Mac 2024 (Microsoft 365 subscription). We performed geographic mapping of all study participants using ArcGIS 10.8 and delineated those who further went forward with one-on-one interviews.

### 2.3. One-on-One Interviews

#### 2.3.1. Recruitment

Oklahoma-based primary care providers were recruited at the end of the online survey as described above, using convenience sampling. Interested participants submitted their contact information, were called, consented, and subsequently scheduled for an interview. Following the interview, they received a gift card.

#### 2.3.2. Interview Conduct

Semi-structured video or telephone interviews were conducted, then audio recordings were de-identified and transcribed. Verbal informed consent was obtained from the participants. Verbal consent was obtained rather than written because of the audio structure of the interview. The interviewer was Kelly Kempe, a Caucasian, female, board-certified vascular surgeon who practiced in Tulsa, Oklahoma. Our team informed the interviewees in advance of the purpose of the study and who the interviewer would be. At the start of each interview, the interviewer again went over the study’s consent, conduct, as well as the interviewer’s role—relaying that their goal is to listen to the PCP’s viewpoints and experiences. All interviews included only the interviewer and the participant. We did not take field notes. The interviews were approximately 45–60 min and included eight open-ended questions (Table 2) to allow for exploration and discourse. In addition to our multidisciplinary research group, OPHIC leadership also edited and approved the interview questions.

### 2.4. Data Transformation

We transcribed each interview from the audio recordings, and each transcript was de-identified and reviewed by two researchers for accuracy. We then downloaded the transcript into MAXQDA software 2022 (VERBI Software 2021) for open coding. The process of assigning codes consisted of at least two researchers independently reading each interview and assigning codes consisting of single words or short phrases that accurately represented the text. Codes were created only from the transcripts. After coding was performed for an interview, codes were reconciled by our multidisciplinary study group (three medical students, a vascular surgeon, and an expert in qualitative data analysis whose clinical practice was internal medicine). The research group created a code book for uniformity, and discussed each code to ensure the given word or phrase fit with the interviewees’ apparent messages. We saved stand-out quotes that highlighted important insight into patients’ and clinicians’ perspectives, and later we reviewed them for additional analysis. Data was continuously analyzed and recoded by the study group at regular meetings in a neutral setting using grounded theory [16]. We applied constant comparison that included practitioner to practitioner, code to new interview, and categories to code. We used descriptive and interpretive techniques to consolidate these codes and identify core variables, which we then coded into themes. The study group found core concepts and processes. From these themes, we categorized the barriers and facilitators to limb preservation. The study group agreed that data saturation had been reached before we coded all interviews. For validity, we also performed a run-length saturation analysis [17].

Our team used multiple tools for qualitative data analysis to visually assess and discuss the evolving themes. Visual tools included hand-written cards of themes to move around and categorize, as well as a whiteboard. MAXQDA’s (2021) frequency and proximity tool and word clouds were used to visualize frequency and proximity, showing specialty care issues, transportation/rurality, and provider education as ongoing challenges and social facilitators and patient education as facilitators. Donabedian’s structure, process, outcome model was applied to help categorize each identified barrier and facilitator and to systematically analyze the factors impacting limb loss risk in a clinical setting. By utilizing and combining these tools in group meetings, we developed final themes and initiated our new theory on barriers and facilitators to limb preservation in Oklahoma.

### 2.5. Data Integration

We integrated the data by comparing the survey and interview results. Areas of convergence and divergence were noted, and the final theory was furthered. Generative artificial intelligence was used after we finalized all qualitative themes, performed and summarized the survey analysis, and created the initial theme. We utilized Microsoft Co-Pilot, 2024, for the purposes of final refinement of the theory and discussion points, to create a barriers and facilitators comparison table, to initiate our final visual model, assist with limitations, and create our graphical abstract. All generated output was reviewed to ensure alignment with the data and to confirm its originality and validity.

## 3. Survey Results

**Figure 1:**
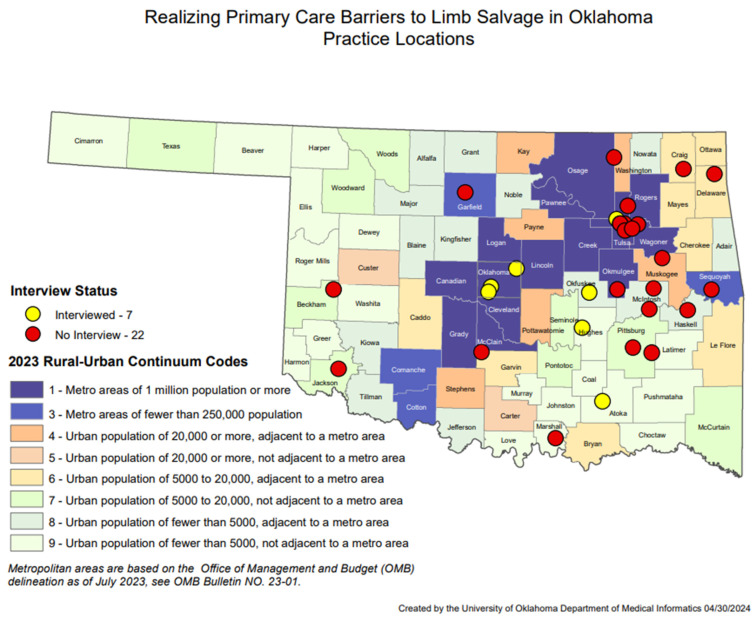
Realizing Primary Care Barriers to Limb Salvage in Oklahoma Practice Locations.

Thirty clinicians from 29 unique primary healthcare clinics in Oklahoma responded to the survey between 2020 and 2021. Of these, 16 (55.2%) were from rural clinics, 13 (44.8%) from urban clinics. One respondent did not provide their clinic address. Figure 1 depicts all practice locations by county and mapped by rural-urban continuum codes. Seventeen clinicians (56.7%) were female, 11 (36.7%) were male, and two (6.7%) did not provide a gender. Twenty-two (73.3%) were White/Caucasian, six (20.0%) were American Indian/Alaska Native, and 2 (6.7%) were Asian. The average age was 55.1 years (standard deviation: 13.5), ranging from 31 to 78 years of age.

Most clinicians (*n* = 29, 96.6%) reported seeing at-risk limbs of amputation (defined as diabetic neuropathy, foot deformities, painful callouses, and claudication) at least every 1–2 months. They saw urgent leg problems (defined as gangrene, non-healing foot wounds, diabetic foot infection, or rest pain) at least once every 6 months (*n* = 25, 83.3%). Most clinicians reported their practices have required annual foot exams for people with diabetes (*n* = 29, 83.0% say often or always) and prescribe antiplatelet medications when applicable for people with diabetes (*n* = 24, 80% saying often or always). Only 35.8% of respondents (*n* = 10) reported providing American Diabetes Association-certified diabetes education when applicable for diabetes. Only 66.7% of respondents (*n* = 20) provide PAD education for affected patients, although 90% (*n* = 27) provide annual visual foot exams and pedal pulse checks for those patients.

Participants without specific protocols already in place unanimously said their practices could benefit from them, including standardized diabetes education protocol (*n* = 17, 56.7% of respondents did not already have this), standing orders for nurses or medical assistants for diabetic patients (*n* = 13, 43.3%), and use of registries or dashboard reviews for diabetic patients (*n* = 18, 60%). Participants also replied they would benefit from including standing orders for nurses or medical assistants for these patients (*n* = 15, 50%), scheduled disease-focused visits from peripheral artery disease (*n* = 11, 37.9%), and use of registries or dashboards for peripheral artery disease (*n* = 19, 63.3%).

Regarding access to and communication with specialists, most clinicians were satisfied or very satisfied with access to podiatry (*n* = 20, 66.7%). Half or less than half of clinicians were satisfied or very satisfied with access to vascular surgery (*n* = 15, 50.0%), vascular ultrasound lab (*n* = 15, 50.0%), interventional specialists (*n* = 13, 43.3%), and endocrinologists (*n* = 12, 40.0%). In Section 7, most clinicians say that after identifying a patient with a limb at risk of amputation, they often or always refer to wound care (*n* = 22, 73.3%) and a vascular specialist (*n* = 21, 70.0%). Other clinicians often or always refer to a general surgeon (*n* = 14, 48.2%), a podiatric surgeon (58.6%), the emergency department (*n* = 9, 30%), or another place (*n* = 2, 20%). Few clinicians received a detailed plan of recommendations from specialists after referral (*n* = 13, 40%), and few relayed initiations of co-management with the specialist after treatment occurs (*n* = 13, 40%).

The health-related social needs most commonly affecting patients with a limb at risk of amputation included (often or always affects) income (*n* = 24, 80%), transportation (*n* = 24, 80%), health insurance (*n* = 21, 70%), and health education (*n* = 20, 66.6%). Issues which are less frequently reported included mental health issues (*n* = 14, 46.7%), substance use disorder (*n* = 13, 43.3%), housing instability (*n* = 11, 36.7%), literacy (*n* = 8, 26.7%), food insecurity (*n* = 6, 20%), racial or ethnic bias (*n* = 5, 17.9%), English language barrier (*n* = 4, 13.3%), and domestic violence (*n* = 1, 3.3%).

Finally, respondents rated initiatives that could improve care for a patient with at-risk limbs on a scale of 1 to 10, with one being most important. Finding vascular specialists in the state willing to travel to their region is ranked 1, 2, or 3 for 47.4% (*n* = 9) of respondents. Implementing an annual foot exam for patients with peripheral artery disease is ranked 8, 9, or 10 for 42.9% (*n* = 9).

## 4. Interview Results

### 4.1. Recruitment

Seven primary care providers were recruited for recorded interviews. All practice locations of the interviewees were mapped in Figure 1, which includes practices located in both metropolitan and rural areas. Five were female (71.4%). The average age was 54 years old. The average time in practice was 20.7 years. All identified as White, and one as White and American Indian. One participant declined to relay their duration of clinical practice, age, or race. Six interviews were conducted from 10 February 2021, to 11 March 2021, and one occurred on 15 October 2021. Two interviewees were physicians with over 10 years of experience as attendings at academic centers with abundant support staff. Five were advanced practice providers (APPs) at rural family practice or internal medicine clinics. One clinic is solely a free clinic for uninsured patients. The other four were described as serving a large portion (>50%) of Medicaid or uninsured patients. The rural clinics all have either a licensed practice nurse or a medical assistant or both as the primary support staff for the provider. Two of the APPs had over 10 years of experience, two had under 5 years of experience, and one did not specify her experience.

### 4.2. Interviews

Themes quickly emerged from the interviews in the form of explicit messages that repeated from one interview to another. Quotations that resonated with the research team are in Table 3, grouped by thematic code.

### 4.3. Interpreting Core Variables and Coding These into Themes

The research team interpreted core variables from the codes into themes, and most themes were categorized as either a barrier or a facilitator to preventing limb loss. Table 4 gives the frequency of these barrier and facilitator themes. “Transportation/Rural Locations” appeared most frequently. We observed in various contexts that when we assigned the transportation theme, primary care providers often noted that accessing specialists is almost always challenging. “Specialty care issues” is a theme that encompasses lack of communication between providers, difficulty in obtaining referrals, poor follow-up after intervention, and confusion about the roles of different specialists. Seven facilitator themes were occurring six or more times. “Peripheral Artery Disease Screening” is a facilitator theme that describes screening methods to prevent amputations, and we frequently saw it within the context of “Specialty Care Issues,” “Patient Education,” “Provider Education,” “Clinic Staff Going Above and Beyond,” and “Lifestyle Interventions”.

Using Donabedian’s structure, process, and outcome framework and applying it to our themes, most themes described by the PCPs fell into the “structure” category, relating to the patient, clinic, and community. We utilized the model, which had the potential to lead towards or away from either the desired primary outcome of reducing amputations or the secondary outcome of improving healthy lives and reducing healthcare costs. Three categories emerged as processes: patient adherence, specialists, and PCP screening.

All qualitative data researchers agreed that the study was saturated prior to the completion of coding. Additionally, a run length analysis was performed, confirming saturation. Zero new codes appeared in the final run (interviews six and seven), which is less than the threshold of 5% of the previously discovered codes.

### 4.4. Integration of Data

Both data sets from the survey and the interviews conveyed consistent data and analysis, indicating a high clinical awareness and occurrence of limbs at-risk, but limited systemic support for limb preservation. The only divergence noted was that most survey respondents said they are satisfied or very satisfied with their access to wound care and podiatry. In contrast, most interviewees suggested access is insufficient. Overall, we assessed that the providers experience an ongoing tension between the barriers and facilitators in amputation prevention. These are summarized in Table 5 and Table 6.

### 4.5. New Theory and Theoretical Model

**Figure 2:**
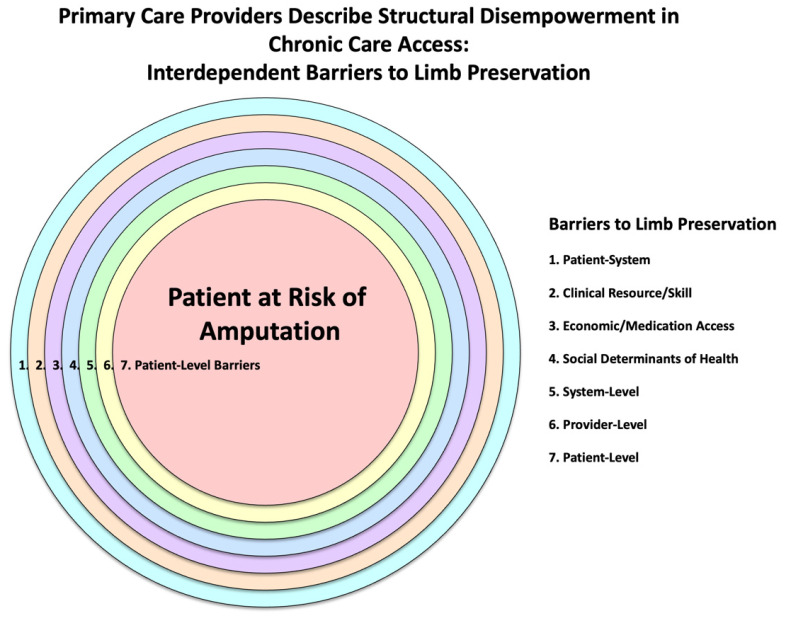
Primary Care Providers Describe Structural Disempowerment in Chronic Care Access: Interdependent Barriers to Limb Preservation.

**Figure 3:**
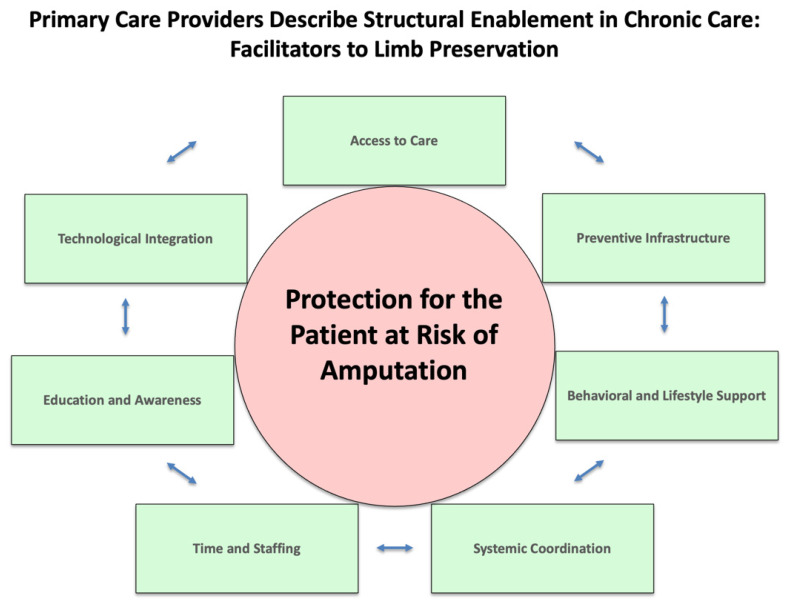
Primary Care Providers Describe Structural Enablement in Chronic Care: Facilitators to Limb Preservation.

Limb preservation is shaped by the tension between structural disempowerment—a compounding network of barriers—and structural enablement—a proactive system of supports. These forces operate across environmental, systemic, provider-level, behavioral, sociocultural, informational, economic, and infrastructural domains, influencing care access, quality, and outcomes. Limb preservation in Oklahoma is contingent upon shifting from structural disempowerment (Figure 2) to structural enablement (Figure 3). This shift requires systemic reform, clinical innovation, and community engagement to build a care environment that is integrated, equitable, and preventive. The convergence of qualitative and quantitative data reveals that empowerment through structure—not just individual behavior—is key to reducing preventable amputations.

## 5. Discussion

This distinctive mixed-method study uncovered that providers believe that patients are systematically disempowered from accessing limb-saving care due to a multi-layered and interdependent network of barriers. PCPs reflected that preventive care is inconsistently implemented, especially in the realms of education and follow-up, and that specialist access and communication are fragmented, contributing to poor continuity. Social determinants remain dominant barriers, especially income, transportation, and insurance.

There are few contemporary studies asking PCPs what barriers there are to chronic disease-related LEA prevention. Ours is the first to be conducted in Oklahoma. Grant et al. recently led a qualitative study involving a group of multidisciplinary healthcare specialists (practicing throughout the United States, with 4 of 18 being PCPs) aimed at understanding barriers to early detection and treatment, as well as factors contributing to disparities in lower extremity PAD [18]. Despite the methodological differences, their study results mirrored ours, finding similar barriers for primary providers that included lack of time, space, guidelines, and education. Additionally, the manuscript highlighted that PAD is challenging to diagnose in a primary care setting using the ankle-brachial index (ABI). Finally, the authors reported that a lack of access to reliable specialists was also common, and additional patient barriers included transportation and education challenges [18]. Another study interviewing 39 healthcare workers who care for diabetic foot ulcers found that care providers located far from each other have more difficulty establishing trusting relationships, which affects quality of communication, timeliness of care, and patient outcomes [19]. We also found that specialty care issues and transportation barriers were routinely compounded together.

Importantly, our findings are consistent with national data and recommendations to improve chronic care. For decades, studies on chronic disease-related LEA have disproportionately demonstrated higher risks of LEA in low-income, minority, and rural groups [20,21,22]. The suggestions offered by primary care providers to address barriers and inequities in limb loss intuitively align with generalizable health disparity recommendations. In particular, the National Institute on Minority Health and Health Disparities (NIMHD) framework, established in 2018, advocates for multi-domain interventions to improve health outcomes among disadvantaged groups [23]. Similarly, the Chronic Care Model has shown success in chronic disease prevention, particularly in diabetes management, through multi-level strategies [24,25]. Policy statements from international and national organizations further reinforce the need for comprehensive, multi-domain approaches to amputation prevention [14]. We employed a mixed-methods and grounded theory design to capture the lived experiences of primary care providers, minimize bias from prior research, and allow themes to emerge organically from the data. Providers described daily challenges and opportunities for improvement that, notably, align with the frameworks of the NIMHD and the Chronic Care Model. We found that the support required for effective amputation prevention spans multiple domains and structural levels. To our knowledge, this is the first study in the lower extremity amputation literature to emphasize the role of structural enablement in reducing amputations related to chronic disease.

### 5.1. Clinical Innovation to Empower Providers

While there is significant variability in the workflow and challenges faced by each clinic, one solution stood out—Provider Education. Interviewees consistently described a perception that they have either missed opportunities to identify patients at risk of limb-loss or they see at-risk patients after severe disease has occurred. As in the study described above, our interviewees also report a lack of equipment or comfort for performing an ABI/PAD screening. Prior literature over 10 years supports that PCPs find ABI tests challenging to perform [26,27]. Recognition of independent predictors of limb amputation is also an important but less-used skill for primary care providers [6]. Though PCPs perceive the ABI test as difficult, there is an educational counterargument. Another study has shown that ABI performed by the primary care provider within 30 days of identification of a diabetic- or PAD-related wound can expedite specialist referral and time to revascularization [28]. This is a compelling reason to educate, empower providers, and initiate ABIs in the clinic. To improve outcomes, time management, and save resources, we recommend a multilayered approach. For instance, consider implementing an EMR search for individuals at risk for PAD. The EMR search can additionally review if the patients with PAD are on guideline directed, best medical therapy. Additionally, create an annual event in September to honor “PAD Awareness Month,” where all patients at risk can undergo screening. Before the awareness month, practitioners may undergo ABI and PAD risk-factor training, utilizing dopplers and cuffs. There is supplemental free educational opportunities for PCPs also offered from the Society for Vascular Surgery, which provides Clinical Practice Guidelines that are relevant to primary care [13,29,30]. Furthermore, the Amputee Coalition has extensive resources for patients at risk of, in preparation for, and after amputation [31]. As one interviewee put it, “can’t lose when you get education to the providers.” 

### 5.2. Systemic Reform and Coordination

Actions to mitigate the limitation to care imposed on patients who live far from specialty services include improving multidisciplinary teams with integrated networks and care coordination. Multidisciplinary limb preservation teams reduce the rate of major amputation [32]. When there is an identified at-risk limb, an urgent consultation to a limb preservation team via telehealth and a direct transfer of care has the opportunity to save tissue and save a limb. For instance, a podiatrist may stop an active infection with debridement, infectious disease will start antibiotics to ensure clearance of bacteremia, and subsequent revascularization to follow by a vascular specialist to save a limb.

Additional ideas that may work for clinics at the primary level include linking diabetes prevention, lifestyle, and vascular protection together as a new program. Additional policy recommendations include promoting a preventive care model over crisis-based interventions, expansion of Medicaid and SoonerCare transportation coverage, capping medication costs, and streamlining assistance programs.

### 5.3. Community Engagement

A patient’s community significantly impacts their adherence, lifestyle, and behaviors, and a lack of social support can have devastating consequences on their health [33]. Our themes found from this study reflect recurring discussions of social support at multiple levels, including clinic staff who leverage strong ties to their community advocates. Quotes from our study highlight staff acting as translators, forming strong relationships, providing emotional support, and going above and beyond for their patients. Community health workers and community-driven educational programs have been successful in improving healthcare outcomes, including in specific culturally tailored healthcare programs, such as diabetes education in Hispanic populations and amputation prevention programs within tribal communities [12,34]. As we consider improvements in Oklahoma, community-based and -led initiatives are imperative.

## 6. Limitations

The response rate to the surveys was lower than expected and may not fully represent the diversity of Oklahoma PCPs, especially across different practice settings and populations served. Data collected between 2020 and 2021 may reflect pandemic-related disruptions in care access, staffing, and patient behavior, which may have influenced provider responses. The low response rate may also reflect widespread understaffing and burnout among PCPs, as documented during this time [35]. Additionally, our sample included a higher proportion of female (57% vs. 37%) and older providers (63% over age 60 vs. 48%) compared to the overall Oklahoma PCP workforce, potentially limiting demographic representativeness [36]. A self-selection bias was also possible with this study design, as participants who opted into the survey and interviews may have been more engaged or interested in limb preservation, potentially skewing results toward more proactive practices or opinions. While saturation was reached in the qualitative interview analysis, additional perspectives may be valuable. While the sample adequately includes both rural (55.2%) and urban (44.8%) clinics—closely reflecting Oklahoma’s rural population distribution (34%)—regional differences in resources, infrastructure, and patient demographics may not be fully captured [36]. The survey relied on self-reported data, which may be subject to recall or social desirability bias. While the integration of our qualitative and quantitative data provided a more comprehensive understanding of provider perspectives on amputation prevention, a more representative sample could yield different results. As we expand this work across Oklahoma and potentially nationwide, we remain committed to inclusive and agile research practices that reflect the full spectrum of provider experiences.

## 7. Conclusions

Oklahoma primary care providers see at-risk feet, witness poor access to care, and desire structural changes to support first-rate preventive care in diabetes and PAD in order to reduce amputations from chronic disease. We theorize limb preservation in Oklahoma is shaped by the tension between structural disempowerment and structural enablement. These forces operate across environmental, systemic, provider-level, behavioral, sociocultural, informational, economic, and infrastructure domains, influencing care access, quality, and outcomes. We identified several intervention strategies to explore, including increasing education for PCPs to empower them to initiate early prevention, improving early identification and preventive therapy for patients at risk for limb loss, and improving specialty care access via networking and policy change. 

## Figures and Tables

**Table 1:** Online Survey Topics.

1	Practice and provider demographics
2	Regularity of diabetes and PAD * leg issues
3	Tests and services used by clinician stratified by diabetes and PAD
4	Protocols used, stratified by diabetes and PAD
5	Who they refer to when there is at-risk limb
6	Social determinants of health that may affect limb preservation
7	Specialty care access and specialty feedback after intervention
8	What practices may need to prevent leg amputation
End of Survey	Respondent asked if they were interested in participating in a one-on-one interview

* Peripheral Artery Disease.

**Table 2:** Semi-structured Interview Questions.

Question 1	To begin, would you mind describing your practice, your clinic, number of practitioners, who your patients are, and the additional support staff you have available?
Question 2	Can you describe, in as much detail as possible, a specific patient case you managed where the patient underwent an above or below-knee amputation due to diabetes, peripheral artery disease, or both?
Question 3	What systems, practices, or strategies do you use or have in place in your clinic to identify patients at risk for amputation?
Question 4	And after these patients are identified, what treatment strategies do you use to reduce your patients’ risk of amputation?
Question 5	What are some of the barriers to caring for patients with diabetes or peripheral artery disease with at-risk limbs that lead to amputation? In other words, why do you think these patients end up with an amputation?
Question 6	What patient or environmental factors do you believe are protective and reduce the risk of amputation in those with DM and PAD?
Question 7	What changes would you like in your own practice?
Question 8	In a perfect world, what do you think would facilitate better care for our patients to reduce limb loss in Oklahoma?

**Table 3:** Significant Interview Quotes Grouped by Relevant Theme.

**Provider and Patient Education**
“People who needed an amputation rarely came through the clinic or the office. They usually ended up with an emergency that was headed in the emergency room or was admitted in the hospital with gangrene or infection or something of that sort. So, I think that’s an important finding here in primary care. We don’t see it! We see the antecedent of it, but I don’t think there was any way we could have predicted who was gonna have a crisis.”
“[We] weren’t really good at separating out claudication pain from uh, neuropathy pain”
“thought of them more as high risk for infection and trauma and never carried it to the ultimate, uh, that that would be—could end up in an amputation”
“You can’t lose when you get education to the providers. You can’t lose. I think that’s probably where it starts, because they’re the ones that’re gonna do your patient education.”
**Specialty Care Issues**
“Obviously, you want to target them before they get there, when they’re right there, and they’re having symptoms of PAD, then the only option really is to do an ER visit.”
“I haven’t been really familiar with any vascular surgeons,”
“Whether it’s with a vascular surgeon, whether it’s with cardiology, I mean, they’ve gotta be able to get there”
“Just establishing network of people around for kind of some support, reaching out, saying hey I’ve got this going on, what do you think? What, what am I missing, what test is there to run uh, um, or who would be best for this patient? Um, it’s probably another kind of good thing to have in this system”
**Outcomes after an amputation**
“We ended up having to put him in the hospital and amputate his leg and he never recovered very effectively from that and ended up dying because he kind of dwindled into despair after that too, so I guess my experience has been that people get despaired when they go through amputation despite my attempts”
**Social facilitators**
“All my MAs are translators. I would bet 60% 65% may be Hispanic”
“My office manager goes above and beyond to try and help these people that really need it. We do go out of our way quite a bit.”

**Table 4:** Most frequently coded barriers and facilitators to limb salvage.

Barriers (Number of Appearances)	Number of Interviews with an Appearance
“Transportation/Rural Locations” (24)	6
“Specialty Care Issues” (23)	7
“Provider Education” (20)	6
“Financial Strain on Patient” (11)	6
“Patient Adherence” (10)	5
“Insurance Barrier” (9)	5
“Patient Lifestyle” (8)	5
“Health Literacy” (8)	3
“Social Inequality” (8)	4
“Navigating Medication Costs” (7)	3
“Lack of Resources for Clinic” (7)	2
**Facilitators (number of appearances)**	**Number of interviews with an appearance**
“Patient Education” (40)	7
“Diagnostic Tests” (21)	7
“Community Facilitator” (15)	5
Clinic Staff Going Above and Beyond (11)	4
“Peripheral Artery Disease Screening” (10)	6
“Provider Education” (7)	4
“Social Facilitator” (6)	3

**Table 5:** Limb Preservation Tension of Final Barriers vs. Facilitators in Oklahoma.

**Structural Disempowerment (Barriers)**	**Structural Enablement (Facilitators)**
Inconsistent providers and fragmented EMRs	Universal EHR and quality dashboards
Limited access to specialists and diagnostics	Access to vascular testing and specialists
Poor transportation and rural isolation	Reliable transport systems and mobile care
Low health literacy and patient misconceptions	Patient education and early outreach
Provider time constraints and lack of training	More time with patients and provider education
Medication cost and navigation burden	Affordable access to essential medications
Housing instability and social inequities	Universal insurance and social support programs
Lack of PAD screening and follow-up	Routine PAD and neuropathy screening
Crisis-based ER care and poor coordination	Electronic treatment algorithms and co-management
Distrust, refusal, and cultural resistance	Dedicated diabetes nurses and culturally tailored messaging

**Table 6:** Primary Care Providers Describe Structural Tension in Limb Preservation in Oklahoma.

**Limb** **Preservation Topic**	**Barrier (Disempowerment)**	**Facilitator (Enablement)**
**EMR**	Inconsistent providers & fragmented EMRs	Universal EHR & quality dashboards
**Specialist**	Limited access to specialists & diagnostics	Access to vascular testing & specialists
**Transportation**	Poor transportation & rural isolation	Reliable transport & mobile care units
**Health Literacy**	Low health literacy & patient misconceptions	Patient education & early outreach
**Capacity**	Provider time constraints & lack of training	More time with patients & provider education
**Medication**	High medication costs & complex navigation	Affordable access to essential medications
**Social Determinants**	Housing instability & social inequities	Universal insurance & social support programs
**PAD Screening**	Lack of PAD screening & follow-up	Routine PAD & neuropathy screening
**Care Coordination**	Crisis-driven ER care & poor coordination	Electronic treatment algorithms & co-management
**Trust & Cultural Fit**	Distrust, refusal, & cultural resistance	Dedicated diabetes nurses & culturally tailored messaging

## Data Availability

The datasets generated and analyzed during this study are not publicly available due to the small number of interview participants and our commitment to preserving confidentiality. Any special requests for access to the data should be directed to Kelly Kempe.

## Supplementary Materials

The following supporting information can be downloaded at: https://www.mdpi.com/article/10.3390/jcm14196817/s1. Survey S1: Realizing Primary Care Barriers to Limb Salvage Survey.

## Abbreviations

LEA	Lower extremity amputation.
PAD	Peripheral artery disease.
PCPs	Primary care providers.
ABI	ankle-brachial index.
