# Supplementary material for: Primary Care Providers Describe Barriers and Facilitators to Amputation Prevention in Oklahoma
Source: J Clin Med. 2025 Sep 26;14(19):6817. doi: 10.3390/jcm14196817 (PMC12525397; doi:10.3390/jcm14196817)
Supplement: Supplementary file 1 [file jcm-14-06817-s001.zip › jcm-3850760-supplementary.pdf]

Realizing Primary Care Barriers to Limb Salvage in Oklahoma

Dear Prospective Participant:

The University of Oklahoma Division of Vascular Surgery in Tulsa and Oklahoma Primary Healthcare Improvement Cooperative (OPHIC) are working to improve vascular health in Oklahoma and study the best strategies to reduce the limb amputation rate in Oklahoma. To better understand the challenges primary care physicians and patients face both in prevention and limb salvage management, we invite your participation in a 5-10 minute online survey.

Your participation in this study is voluntary. There is no penalty if you choose not to participate. You can withdraw at any time. While your name, email, and practice location will be known, all data will securely kept in password proteted REDCap, be deidentified prior to analysis, and only aggregate demographic data will be seen by those outside the research team.

All efforts will be made to keep your information secure. There is no direct benefit of the study. We hope that your participation will contribute toward a better understanding of limb salvage management and could increase the availability of interventions/programs throughout Oklahoma.

Completion of this survey will be considered your consent to participate. After completing the survey, you have the option to enter a random drawing for one $100 Walmart gift card.

If you have any questions concerning the research study or would like to have your results withdrawn at any time, please contact the Principal Investigator, Kelly Kempe at kelly-kempe@ouhsc.edu or (918) 634-7560 or (206)-779-6646. You can also contact our Research Coordinator, Carol Cox at Carol-Cox@ouhsc.edu. For questions about your rights as a participant, contact the OUHSC Director of the Human Research Participant Program at (405) 271-2045.

The University of Oklahoma is an equal opportunity institution. IRB #12773.

I am a current primary healthcare provider in Oklahoma (MD, DO, NP, or PA).

◯ Yes
◯ No

What is the practice name and address are you answering this survey about?

Practice Name ________________________________

Address ________________________________

Address 2 ________________________________

City ________________________________

State ________________________________

Zip Code ________________________________

1. Our practice sees patients who are at risk for leg amputation with . . .

	Never	Once a Year	Once Every 6 Months	Once Every 1-2 Months	One or More Times per Week
non-urgent leg problems (diabetic neuropathy, foot deformities, painful callouses, claudication).	◯	◯	◯	◯	◯
urgent leg problems (open non-healing foot wounds, infection, gangrene, rest pain).	◯	◯	◯	◯	◯

2. Please indicate for each item below how frequently your clinic performs this test or service for patients with diabetes mellitus (Type 1 or Type 2) . . .

	Never	Rarely	Sometimes	Often	Always
annual visual foot exam.	◯	◯	◯	◯	◯
annual pedal pulse.	◯	◯	◯	◯	◯
annual sensory foot exam with mono filament.	◯	◯	◯	◯	◯
annual counseling for patients with diabetes mellitus warning of possible foot problems.	◯	◯	◯	◯	◯
HgbA1c testing per ADA recommendations.	◯	◯	◯	◯	◯
smoking cessation counseling and intervention when applicable.	◯	◯	◯	◯	◯
antiplatelet medication when applicable.	◯	◯	◯	◯	◯
statin medication when applicable.	◯	◯	◯	◯	◯
diabetes mellitus education delivered by an ADA certified educator when applicable.	◯	◯	◯	◯	◯
cardiovascular event risk assessment (ASCVD), Framingham, Reynolds, etc.)	◯	◯	◯	◯	◯

3. For patients with absent pedal pulses or other evidence of peripheral arterial disease, please indicate for each item below how frequently your clinic performs this test or service . . .

	Never	Rarely	Sometimes	Often	Always
annual visual foot exam.	○	○	○	○	○
annual pedal pulse.	○	○	○	○	○
annual sensory foot exam with mono filament.	○	○	○	○	○
annual counseling for patients with peripheral arterial disease warning of possible foot problems.	○	○	○	○	○
smoking cessation counseling and intervention when applicable.	○	○	○	○	○
antiplatelet medication unless contraindicated.	○	○	○	○	○
statin medication when applicable.	○	○	○	○	○
provide peripheral arterial disease education.	○	○	○	○	○

Please help us better understand the protocols for diabetes mellitus currently used in your practice.

4. Protocols for patients with diabetes mellitus (Type 1 or Type 2) to identify legs at risk such as . . .

	I see no need	Our practice has already	Our practice could benefit
standardized diabetes education protocol.	◯	◯	◯
standardized diabetes visits.	◯	◯	◯
standing orders for nurses/MAs.	◯	◯	◯
scheduled disease focused visits.	◯	◯	◯
use of registries or dashboard reviews.	◯	◯	◯

Please help us better understand the protocols for peripheral arterial disease currently used in your practice.

5. Protocols for patients with peripheral arterial disease to identify legs at risk such as . . .

	I see no need	Our practice has already	Our practice could benefit
standardized diabetes education protocols.	○	○	○
standardized diabetes visits.	○	○	○
standing orders for nurses/MAs.	○	○	○
scheduled disease focused visits.	○	○	○
use of registries or dashboard reviews.	○	○	○

6. In your practice setting, how satisfied are you with access to and reports of evaluation for the following services?

	Very Dissatisfied	Dissatisfied	Neutral	Satisfied	Very Satisfied
Vascular Surgery	◯	◯	◯	◯	◯
Interventional Specialist (Interventional Radiology or Cardiology)	◯	◯	◯	◯	◯
Podiatry	◯	◯	◯	◯	◯
Endocrinology (Diabetology)	◯	◯	◯	◯	◯
Vascular Ultrasound Diagnostic Lab	◯	◯	◯	◯	◯

7. When I identify a patient with an urgent, limb threatening medical problem such as gangrene, rest pain, infection, or non-healing foot ulcer, I refer to . . .

	Never	Rarely	Sometimes	Often	Always
wound care.	○	○	○	○	○
a general surgeon.	○	○	○	○	○
a vascular specialist.	○	○	○	○	○
a podiatric surgeon.	○	○	○	○	○
the emergency department.	○	○	○	○	○
other (please specify).	○	○	○	○	○

Other, please specify

____

8. After a patient undergoes treatment for a leg at risk . . .

	Never	Rarely	Sometimes	Often	Always
I receive a detailed plan with recommendations and plan to implement after treatment occurs.	◯	◯	◯	◯	◯
Co-management is initiated between our practice and the specialist after treatment occurs.	◯	◯	◯	◯	◯

9. Many patients struggle with health related social needs. We are interested in your patient population. When considering patients with a diagnosis of DM/PAD with legs at risk for an amputation (gangrene, rest pain, infection, or non-healing foot ulcer), how often do your patients have challenges associated with the following health related social needs?

	Never	Rarely	Sometimes	Often	Always
Biases associated with race and/or ethnicity	○	○	○	○	○
Income	○	○	○	○	○
Health education	○	○	○	○	○
Health insurance	○	○	○	○	○
Transportation	○	○	○	○	○
Substance use disorder	○	○	○	○	○
Food insecurity	○	○	○	○	○
Mental health issues	○	○	○	○	○
Housing instability	○	○	○	○	○
Domestic violence	○	○	○	○	○
Literacy	○	○	○	○	○
English language difficulty	○	○	○	○	○
Other (please specify)	○	○	○	○	○

Other

____

10. Our practice's priorites to improve the care for DM/PAD patients for limb salvage include: (Please rank in order of importance)

	1-Most Important	2	3	4	5	6	7	8	9	10-Least Important
Finding vascular specialists in the state willing to travel to our region	○	○	○	○	○	○	○	○	○	○
Using telehealth resources for vascular consults	○	○	○	○	○	○	○	○	○	○
Refer recalcitrant smokers to a program that works	○	○	○	○	○	○	○	○	○	○
Adopting a co-management system for hard to control diabetic patients	○	○	○	○	○	○	○	○	○	○
Implementing an annual foot exam for diabetics	○	○	○	○	○	○	○	○	○	○
Implementing an annual foot exam for patients with peripheral arterial disease	○	○	○	○	○	○	○	○	○	○
Adopting affordable medications to promote adherence	○	○	○	○	○	○	○	○	○	○
Budgeting time to provide DM and nutritional counseling	○	○	○	○	○	○	○	○	○	○
Engaging specialists to help with interdisciplinary care such as nutrition, medication management, or education	○	○	○	○	○	○	○	○	○	○
Creating a registry to better track our patients and their health needs	○	○	○	○	○	○	○	○	○	○

11. What additional items would you like to see happen to improve the care for DM/PAD patients to prevent leg amputation? (Please type you answers in the space below.)

Demographics

These next questions will help us understand the practitioner who took this survey. The public will never have access to individual answers to the survey.

12. What is your role in this practice?
    - ◯ DO
    - ◯ MD
    - ◯ NP
    - ◯ PA
    - ◯ Other (please explain)

Other, please describe: ______________________________

13. Years practicing in primary care: ______________________________

14. What best describes your gender?
    - ◯ Male
    - ◯ Female
    - ◯ Prefer not to answer
    - ◯ Prefer to self describe (please explain)

Other, please describe: ______________________________

15. What is your age? ______________________________

16. What is your race?
    - ☐ American Indian or Alaska Native
    - ☐ Asian or Asian American
    - ☐ Black or African American
    - ☐ Native Hawaiian or Pacific Islander
    - ☐ White
    - ☐ Other
    - ☐ Prefer not to answer

17. Are you of Hispanic, Latino, or Spanish origin?
    - ◯ Yes
    - ◯ No
    - ◯ Prefer not to answer

18. Are you interested in further participation on this topic of limb salvage by participating in a recorded zoom interview?
    - ◯ Yes
    - ◯ No

First Name: ______________________________

Last Name: ______________________________

Please include an email address for contact for the audio/visual recorded zoom meeting ______________________________

Please list a contact phone number. ______________________________
